# Supplementary material for: Translational control of polyamine metabolism by CNBP is required for Drosophila locomotor function
Source: eLife. 2021 Sep 14;10:e69269. doi: 10.7554/eLife.69269 (PMC8439652; doi:10.7554/eLife.69269)
Supplement: Figure 6—figure supplement 2—source data 1. [file elife-69269-fig6-figsupp2-data1.docx]

| **Position (bp)** | **Motif sequence** | **Transcript sequence** | **Z score** | **P value** |
| --- | --- | --- | --- | --- |
| 89 | uggagnw | cgcgagcucaacauccgccggguga**ucgagga**gugcgaccugcagcgccuggaccag | 3.048 | 1.15e-03 |
| 113 | uggagnw | aucgaggagugcgaccugcagcgcc**uggacca**ggcccucaacaucugcgaccugucu | 2.655 | 3.97e-03 |
| 149 | uggagnw | cucaacaucugcgaccugucuagcg**uggagcg**uaagcugcgccucuggcagaagcuc | 2.226 | 1.30e-02 |
| 230 | uggagnw | gccgucaagugcaaugacgauccaa**ugguggu**caggcugcuggcccagcugggagcc | 2.226 | 1.30e-02 |
| 365 | uggagnw | aaucccugccgcccugucagccauc**uggagua**cgccaaggagcaccagguguccaac | 1.905 | 2.84e-02 |
| 377 | uggagnw | ccugucagccaucuggaguacgcca**aggagca**ccagguguccaacggaacgguggac | 2.548 | 5.42e-03 |
| 404 | uggagnw | gagcaccagguguccaacggaacgg**uggacaa**ugaguucgagguauacaagcugcac | 2.655 | 3.97e-03 |
| 416 | uggagnw | uccaacggaacgguggacaaugagu**ucgaggu**auacaagcugcacacgcacuauccc | 2.655 | 3.97e-03 |
| 569 | uggagnw | gcccuaaugcugcuggccaaauccu**uggagcu**gaaggugaccggcaccaguuuccac | 3.500 | 2.33e-04 |
| 575 | uggagnw | augcugcuggccaaauccuuggagc**ugaaggu**gaccggcaccaguuuccacgucggc | 3.071 | 1.07e-03 |
| 695 | uggagnw | uucggcgcacuacugggcuaugaca**uggacuu**ucuggacauuggcgguggguucccu | 3.131 | 8.71e-04 |
| 704 | uggagnw | cuacugggcuaugacauggacuuuc**uggacau**uggcgguggguucccuggcagcgau | 2.869 | 2.06e-03 |
| 711 | uggagnw | gcuaugacauggacuuucuggacau**uggcggu**ggguucccuggcagcgaugacguaa | 2.869 | 2.06e-03 |
| 746 | uggagnw | uucccuggcagcgaugacguaaagu**uugagaa**gauagccgaaagugugaauaccucg | 2.226 | 1.30e-02 |
| 881 | uggagnw | uugguuugcaagauccacgccaagc**gggagau**caggaacgaagcuggcaaacuggac | 2.226 | 1.30e-02 |
| 980 | uggagnw | aacugcauucuguacgaccaucaag**uggugau**ugcagagcauuaucuggacaaugca | 2.917 | 1.77e-03 |
| 1001 | uggagnw | caaguggugauugcagagcauuauc**uggacaa**ugcagaaucuuugccacaccuaaag | 3.131 | 8.71e-04 |
| 1008 | uggagnw | ugauugcagagcauuaucuggacaa**ugcagaa**ucuuugccacaccuaaaguccuuga | 2.655 | 3.97e-03 |
| 1076 | uggagnw | aguugugacgcccuagauaagauuu**cggagga**ccugcacuugcccaaccuaaaccga | 2.226 | 1.30e-02 |
