# Supplementary material for: Translational control of polyamine metabolism by CNBP is required for Drosophila locomotor function
Source: eLife. 2021 Sep 14;10:e69269. doi: 10.7554/eLife.69269 (PMC8439652; doi:10.7554/eLife.69269)
Supplement: Source data 1. [file elife-69269-data1.pptx]

## Slide 1
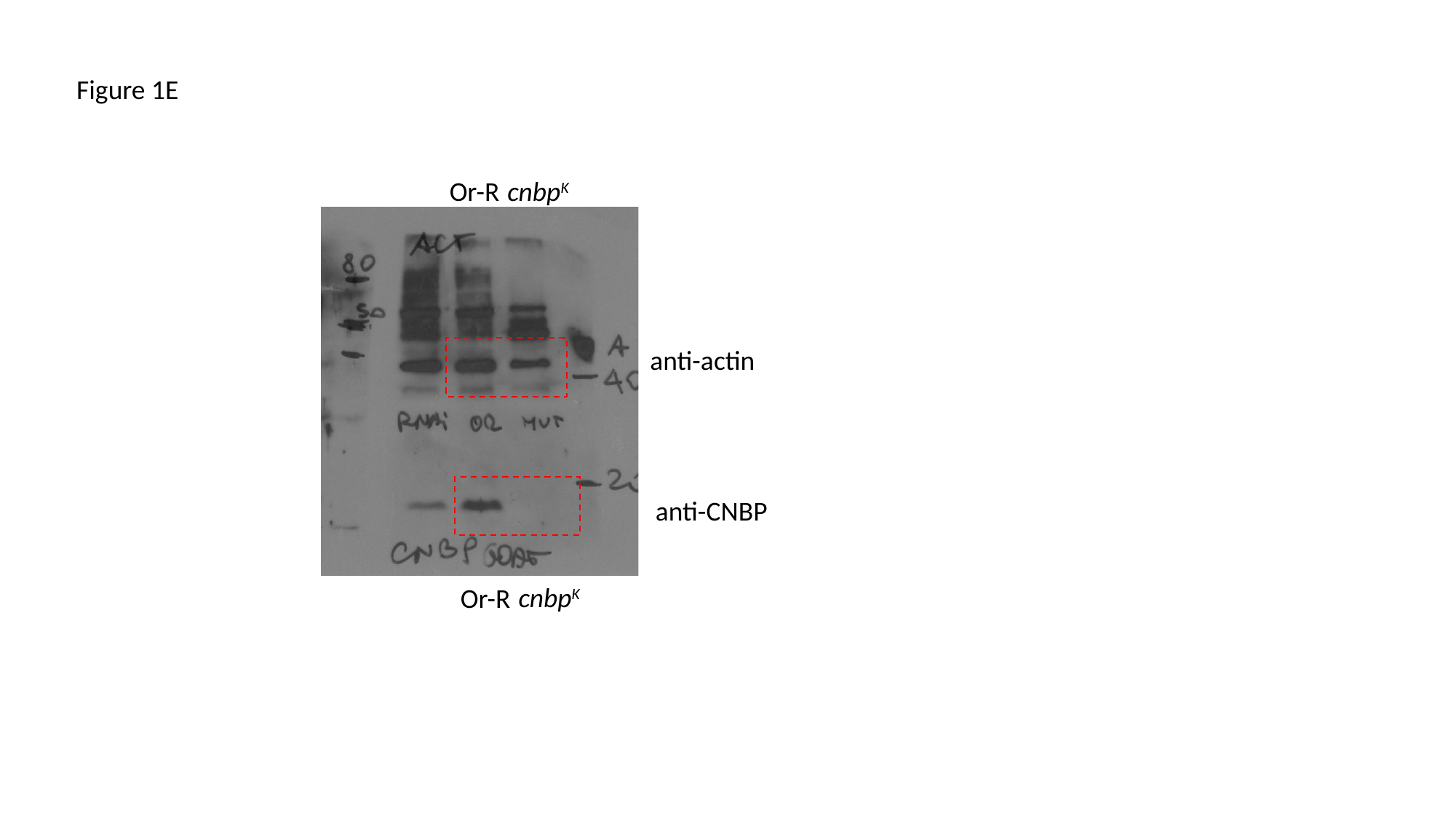

Figure 1E
Or-R
cnbpK
anti-actin
anti-CNBP
cnbpK
Or-R

## Slide 2
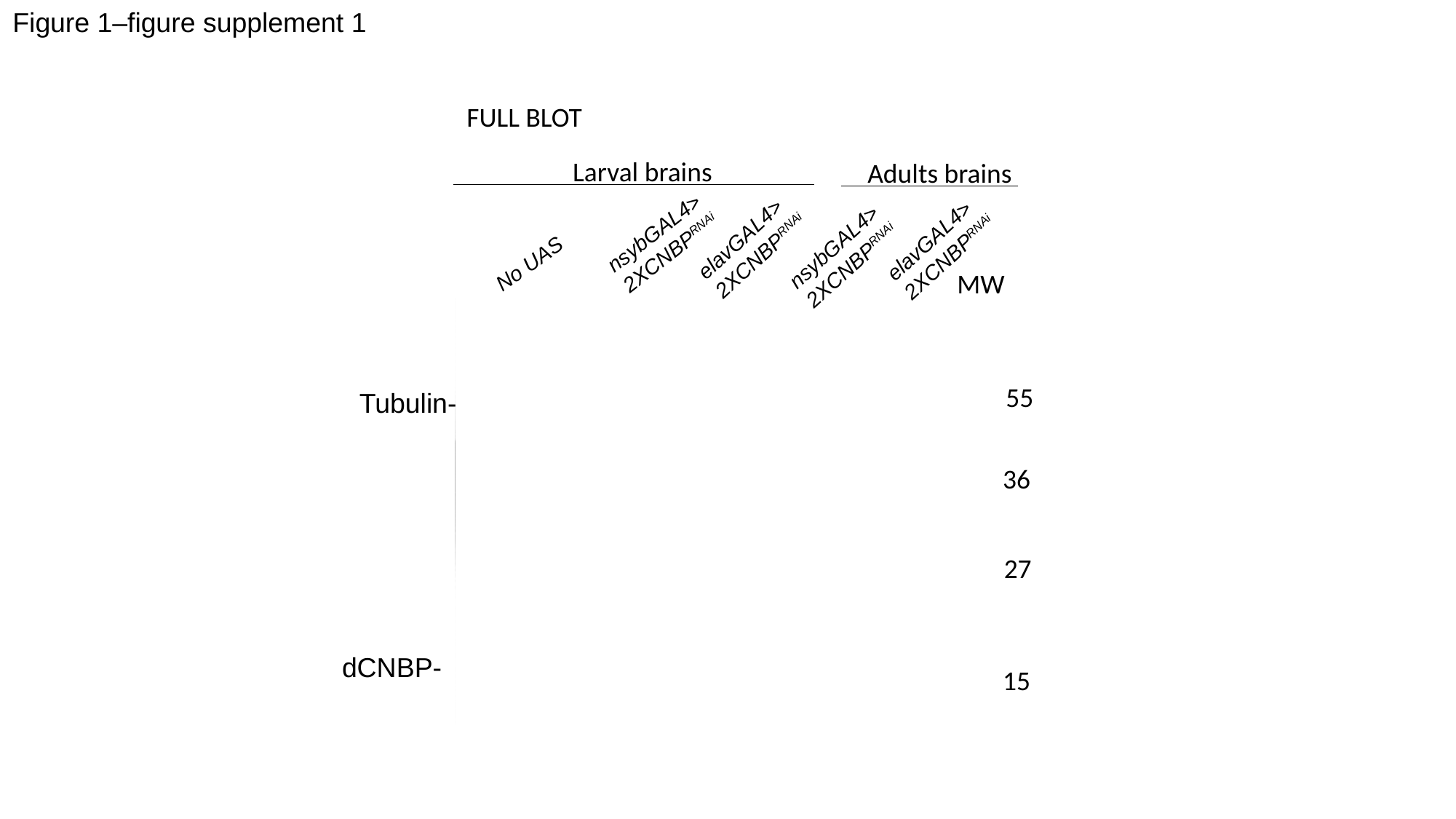

Figure 1–figure supplement 1
FULL BLOT
Larval brains
Adults brains
nsybGAL4>
2XCNBPRNAi
elavGAL4>
2XCNBPRNAi
elavGAL4>
2XCNBPRNAi
nsybGAL4>
2XCNBPRNAi
No UAS
MW
55
Tubulin-
36
27
dCNBP-
15

## Slide 3
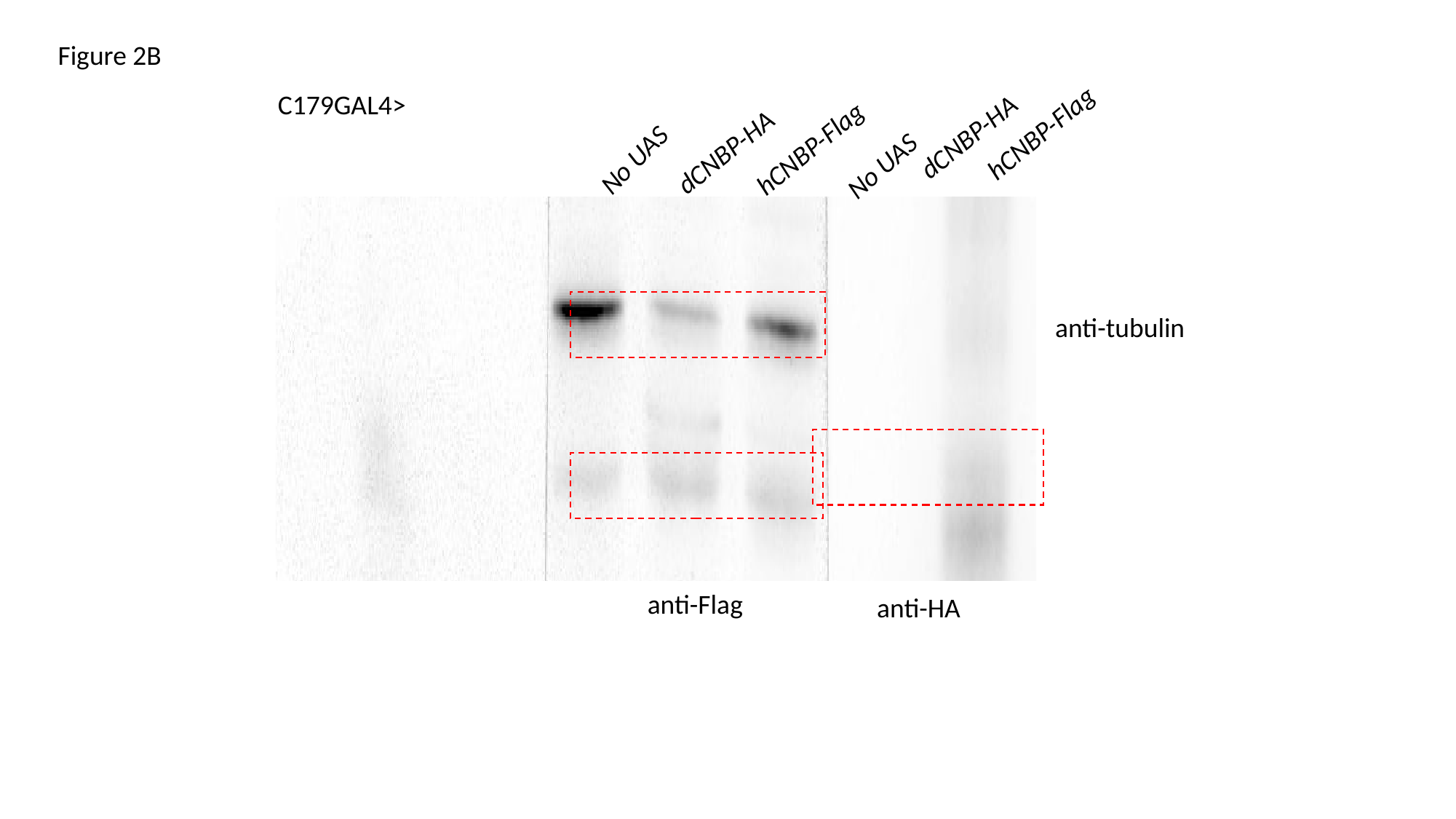

Figure 2B
C179GAL4>
hCNBP-Flag
dCNBP-HA
hCNBP-Flag
dCNBP-HA
No UAS
No UAS
WT
WT
Anti-Tub
anti-tubulin
anti-Flag
anti-HA

## Slide 4
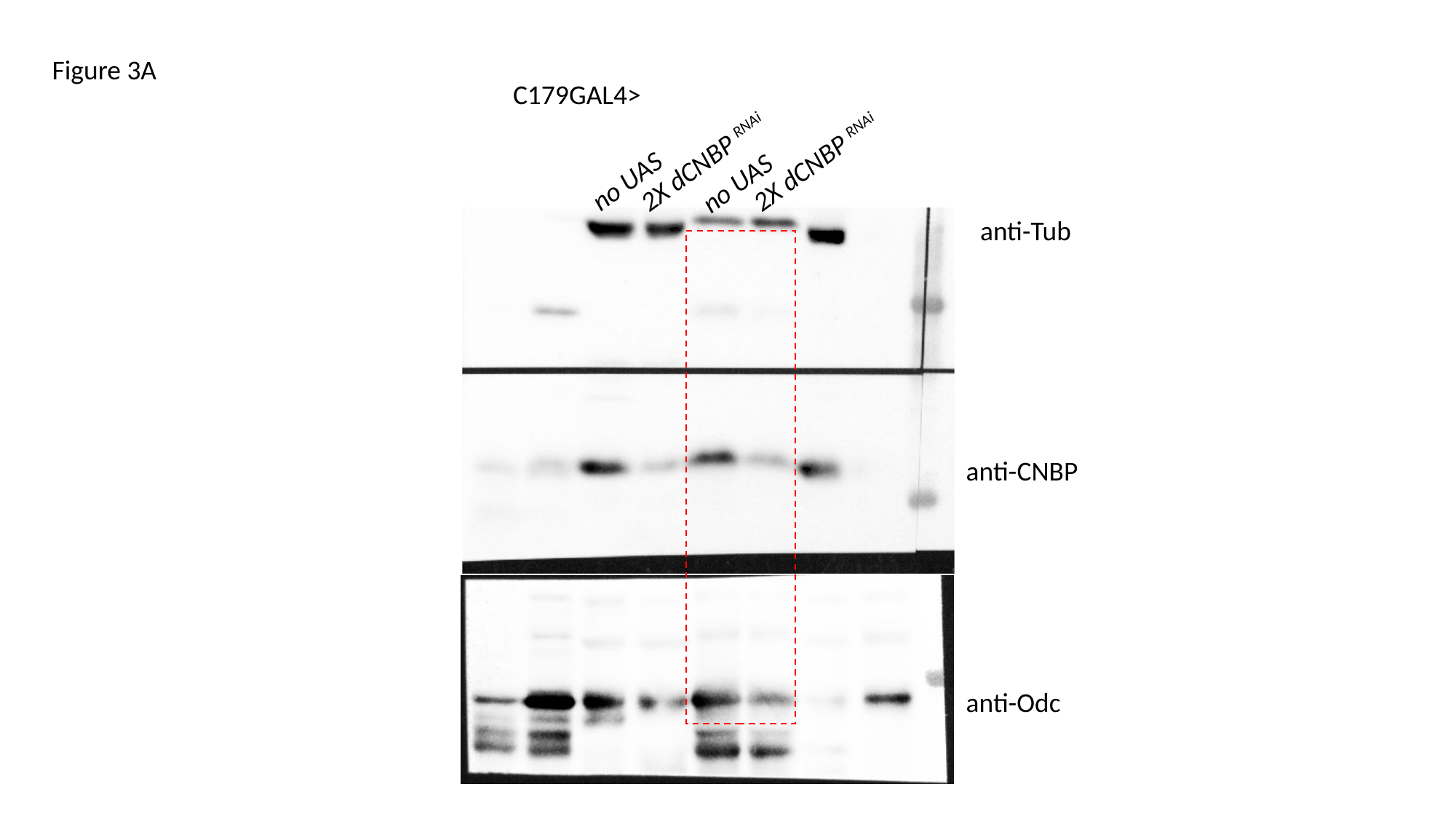

Figure 3A
C179GAL4>
2X dCNBP RNAi
2X dCNBP RNAi
no UAS
no UAS
anti-Tub
anti-CNBP
anti-Odc

## Slide 5
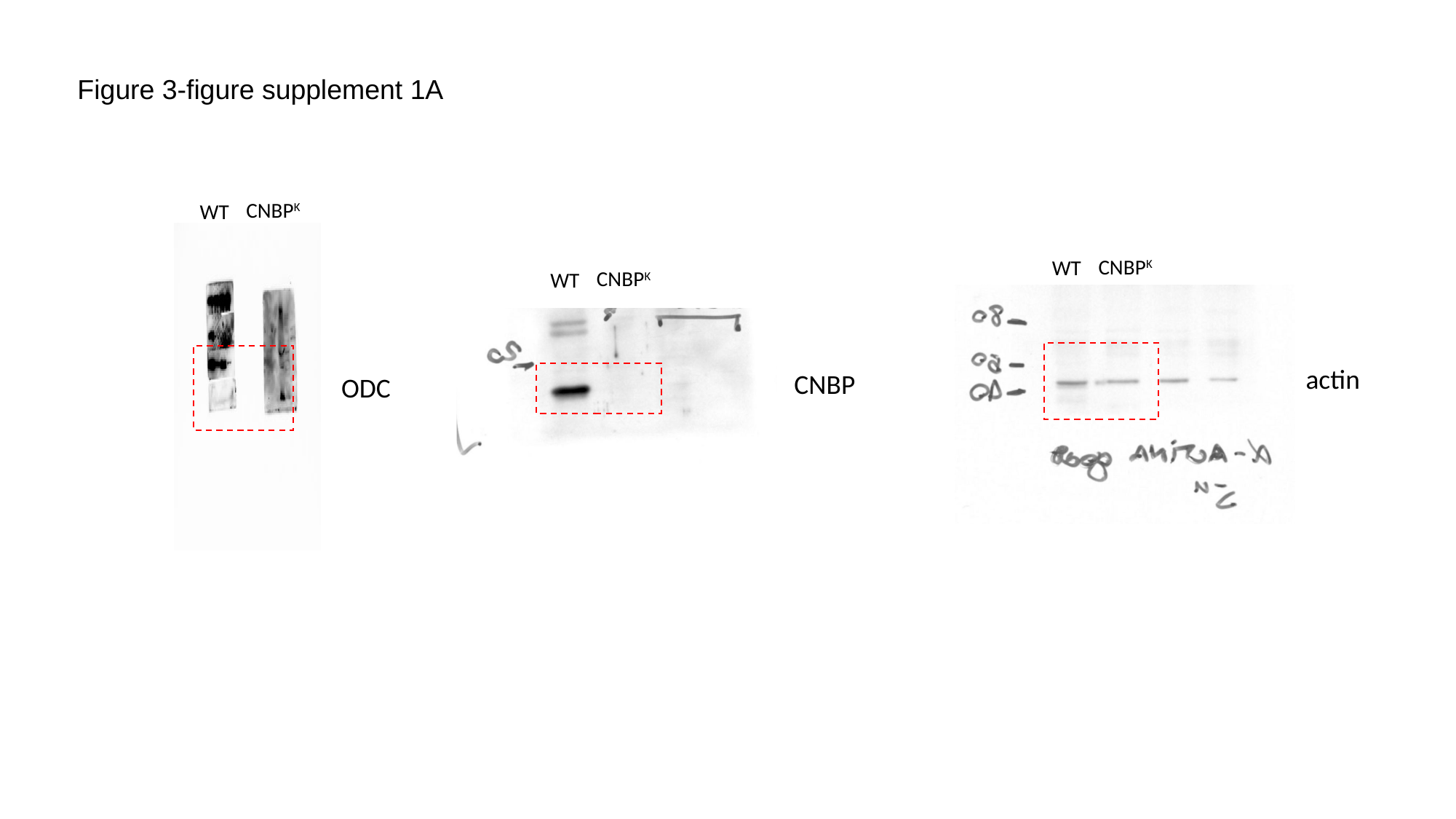

Figure 3-figure supplement 1A
CNBPK
WT
CNBPK
WT
CNBPK
WT
actin
CNBP
ODC

## Slide 6
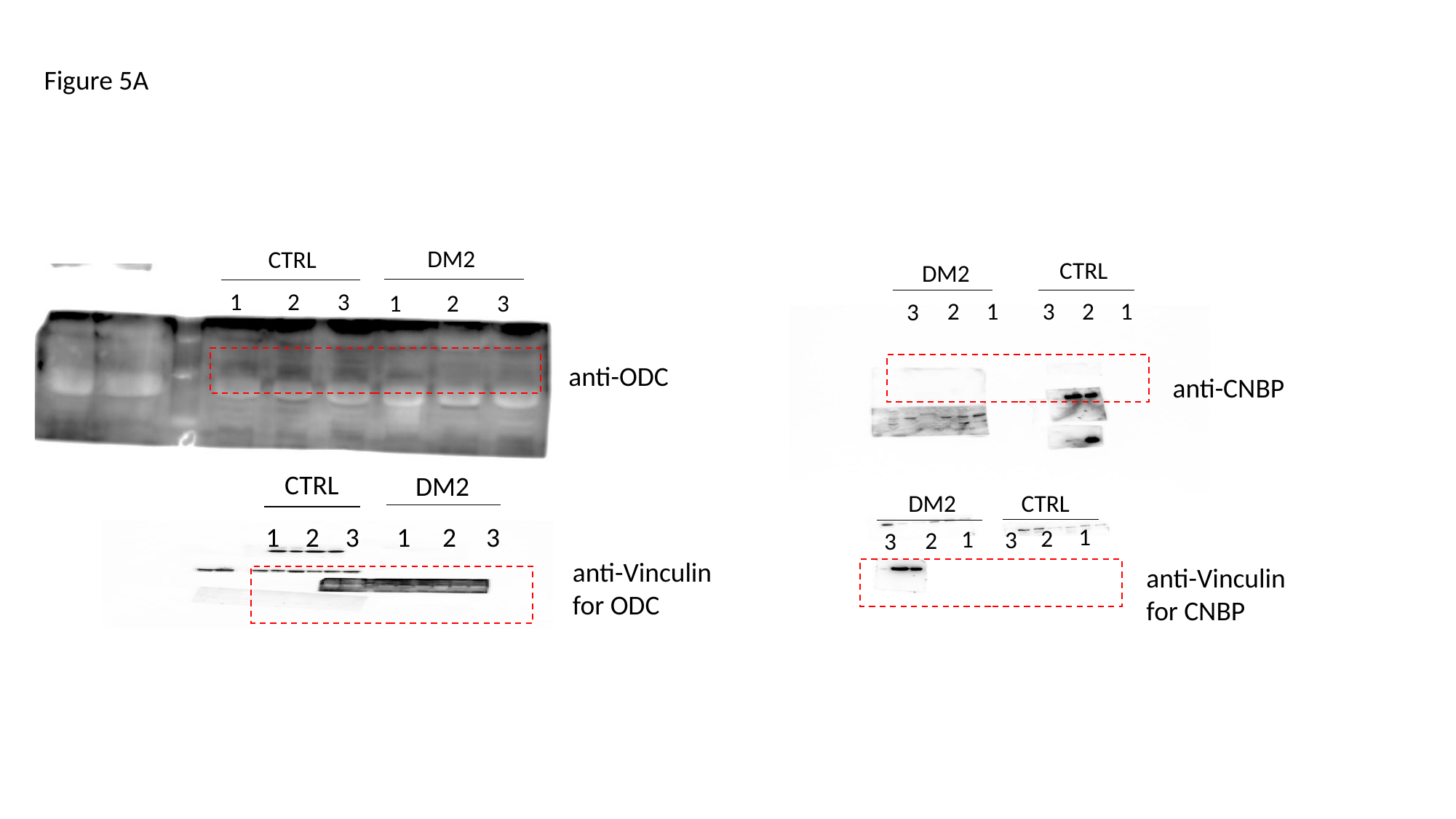

Figure 5A
DM2
CTRL
CTRL
DM2
1
2
3
1
2
3
2
1
3
2
1
3
anti-ODC
anti-CNBP
CTRL
DM2
CTRL
DM2
1
2
3
1
2
3
1
2
1
3
2
3
anti-Vinculin
for ODC
anti-Vinculin
for CNBP

## Slide 7
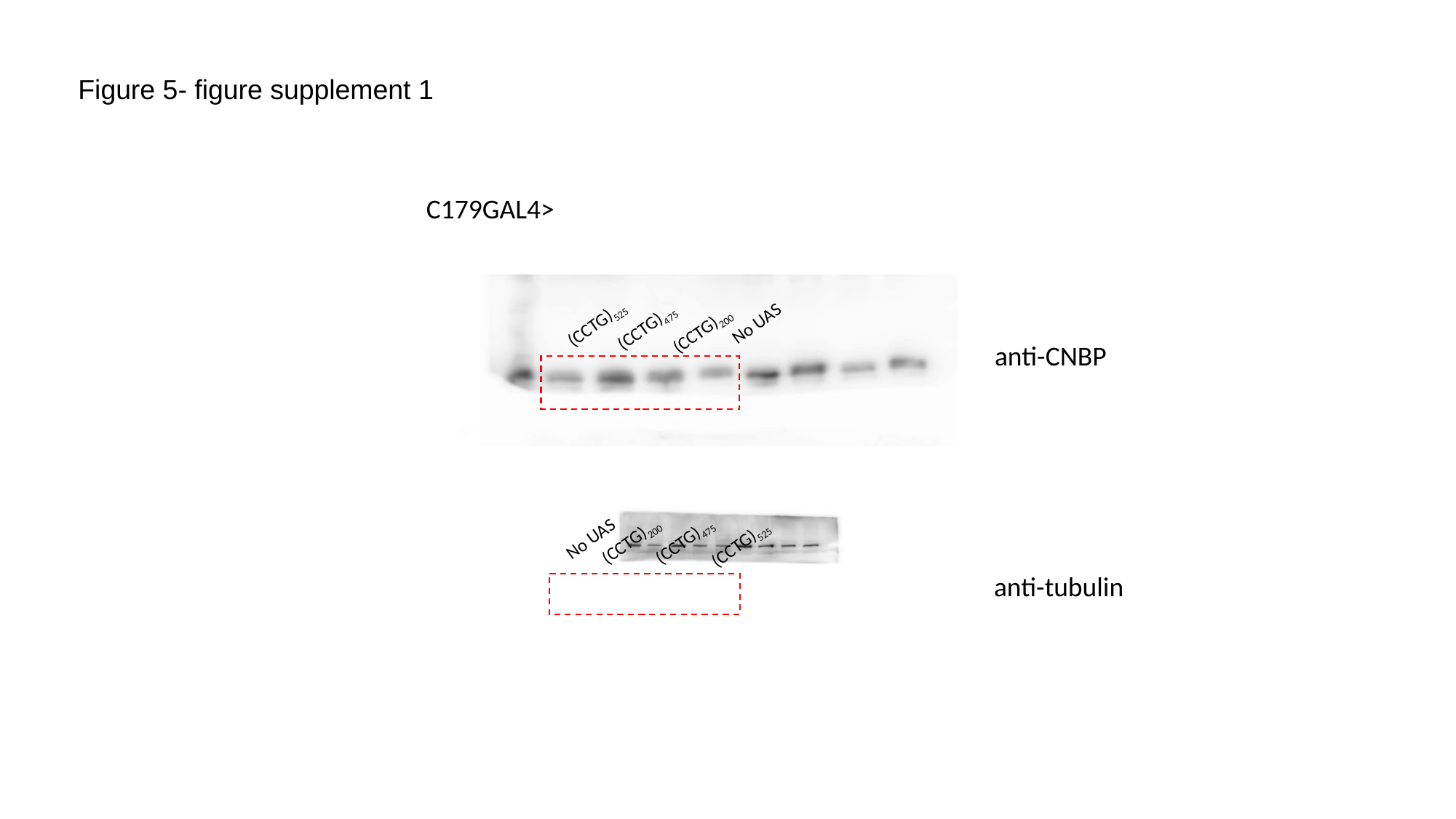

Figure 5- figure supplement 1
C179GAL4>
(CCTG)475
(CCTG)200
No UAS
(CCTG)525
anti-CNBP
(CCTG)200
(CCTG)475
No UAS
(CCTG)525
anti-tubulin

## Slide 8
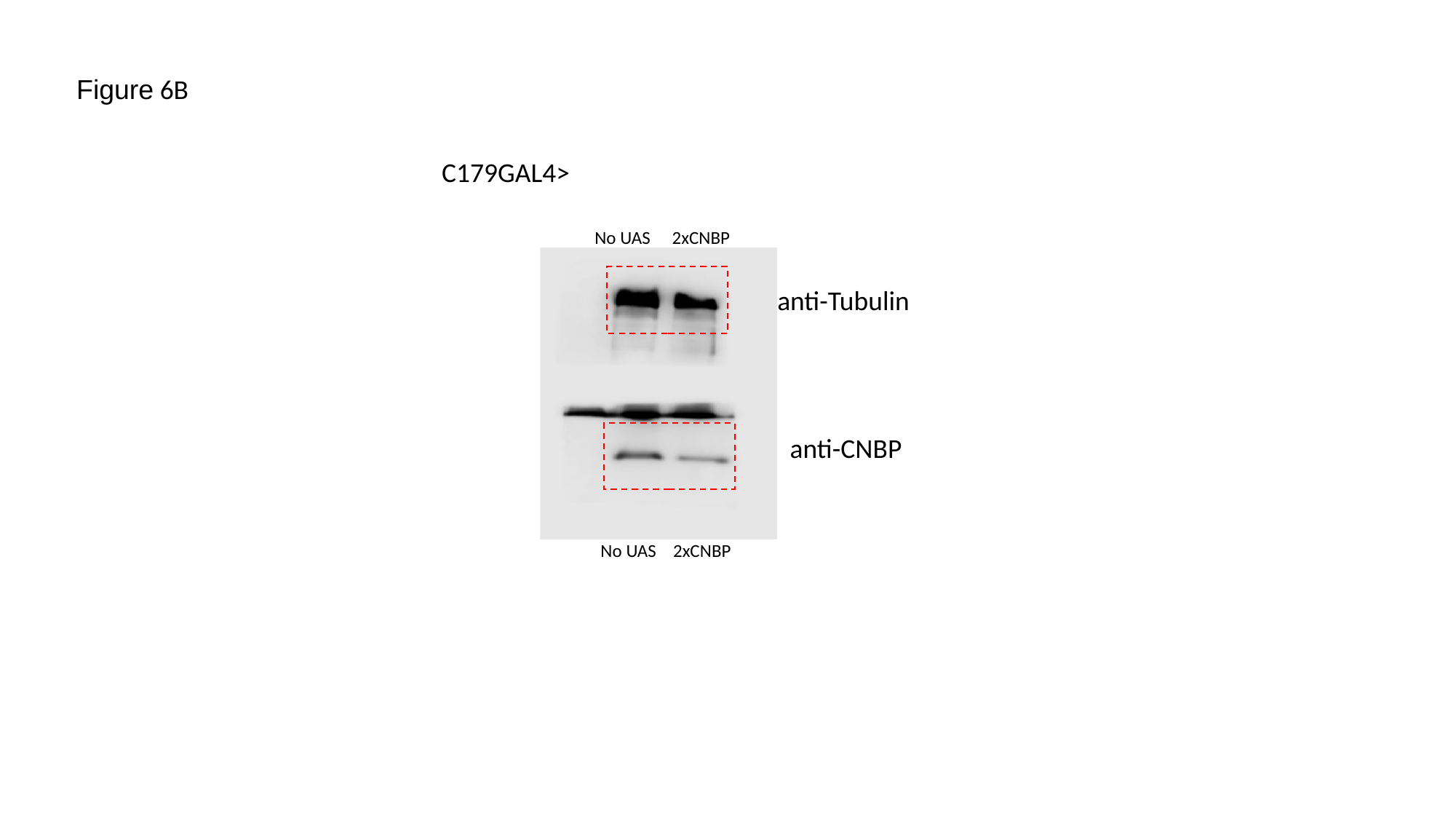

Figure 6B
C179GAL4>
2xCNBP
No UAS
anti-Tubulin
anti-CNBP
2xCNBP
No UAS

## Slide 9
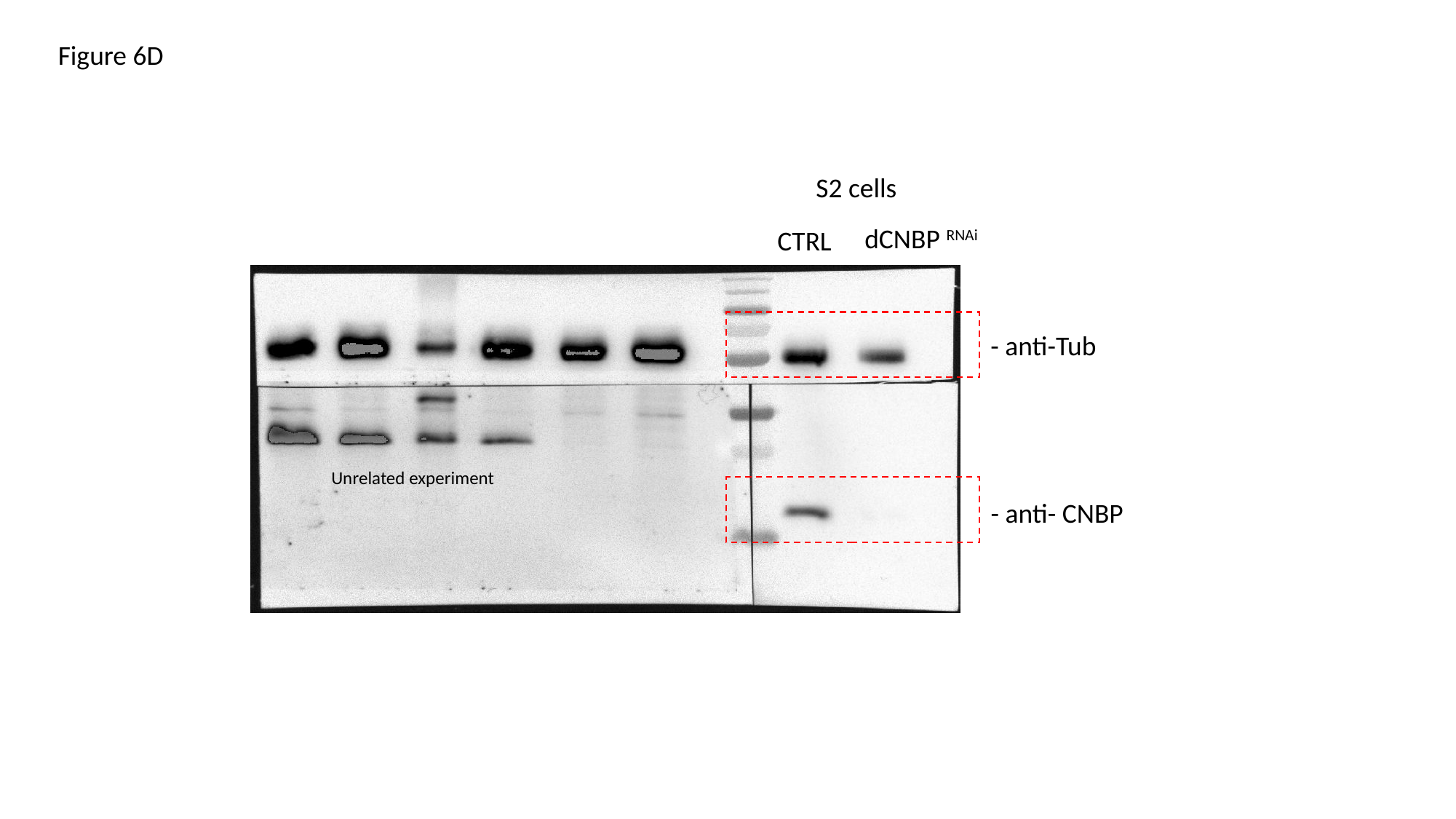

Figure 6D
S2 cells
dCNBP RNAi
CTRL
- anti-Tub
Unrelated experiment
- anti- CNBP

## Slide 10
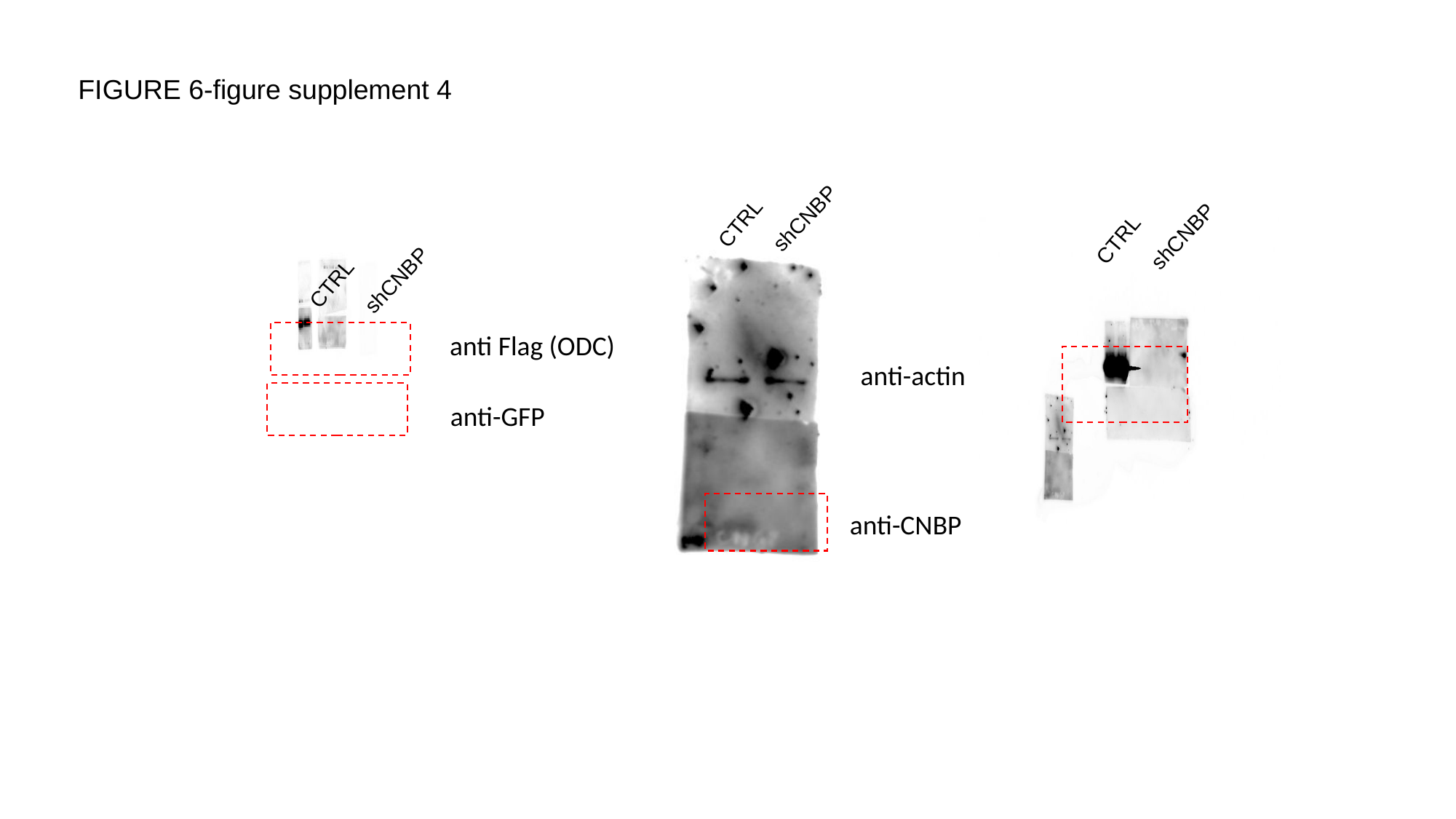

FIGURE 6-figure supplement 4
shCNBP
CTRL
shCNBP
CTRL
shCNBP
CTRL
anti Flag (ODC)
anti-actin
anti-GFP
anti-CNBP

## Slide 11
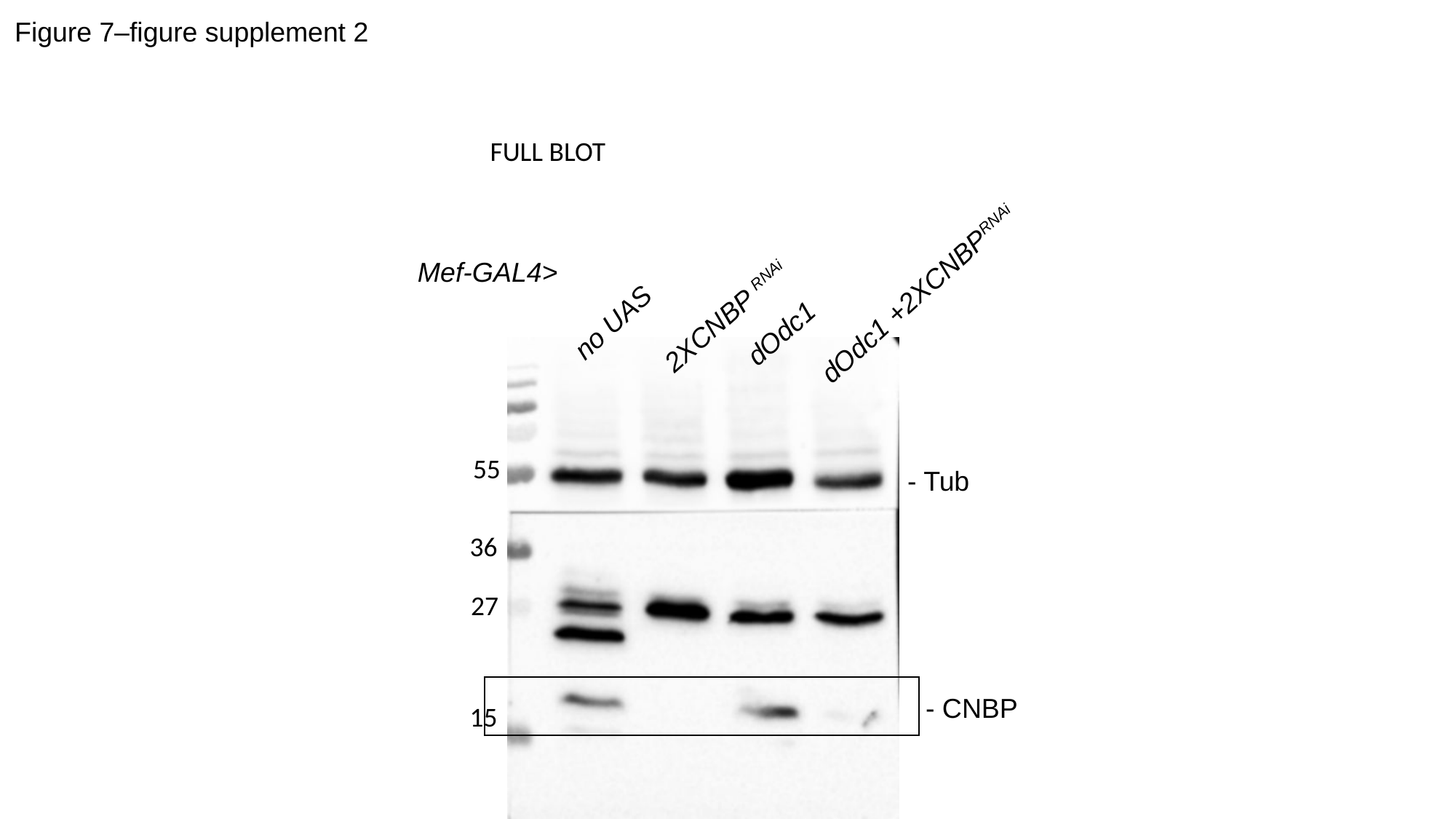

Figure 7–figure supplement 2
FULL BLOT
2XCNBP RNAi
Mef-GAL4>
dOdc1 +2XCNBPRNAi
no UAS
dOdc1
55
- Tub
36
27
- CNBP
15
