# Supplementary figures and images for: Translational control of polyamine metabolism by CNBP is required for Drosophila locomotor function
Source: eLife. 2021 Sep 14;10:e69269. doi: 10.7554/eLife.69269 (PMC8439652; doi:10.7554/eLife.69269)

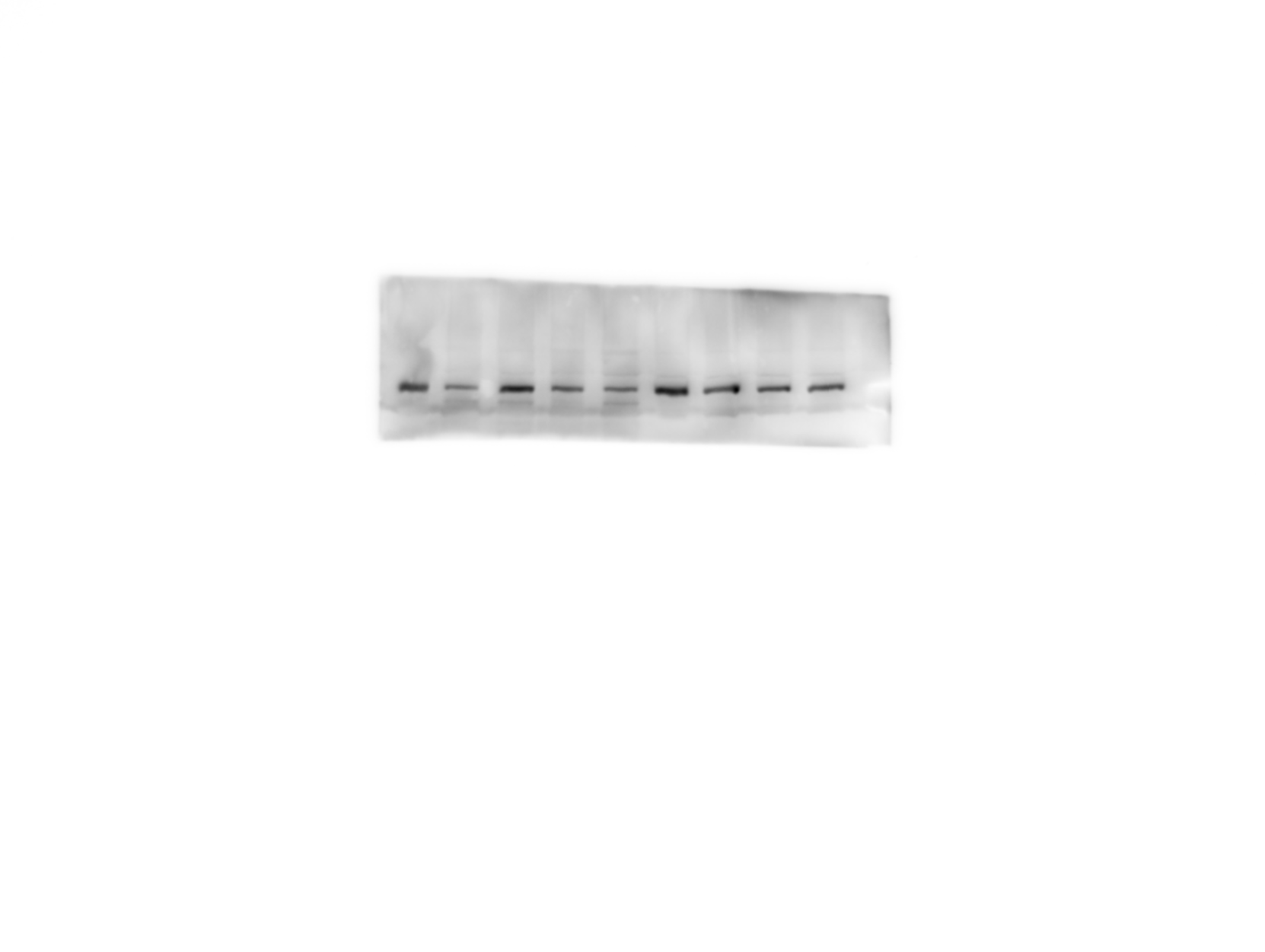

Supplement: Source data 2. [file elife-69269-data2.zip › tiff blots Fig1-7/Fig5-S1 source data 2b.tif]

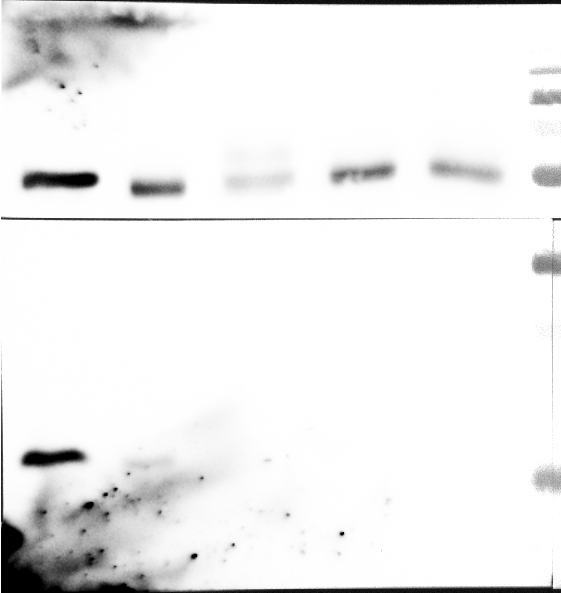

Supplement: Source data 2. [file elife-69269-data2.zip › tiff blots Fig1-7/Fig1-S1source data.tif]

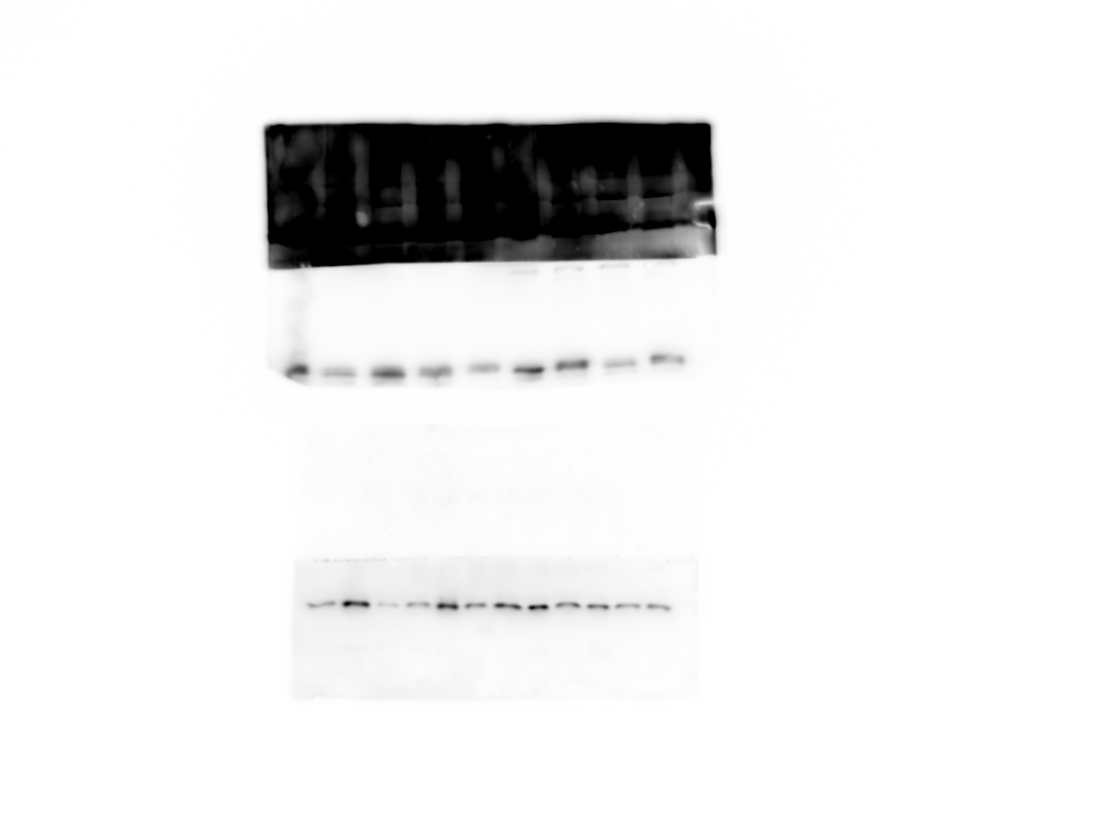

Supplement: Source data 2. [file elife-69269-data2.zip › tiff blots Fig1-7/Fig5-S1 source data 2a.tif]

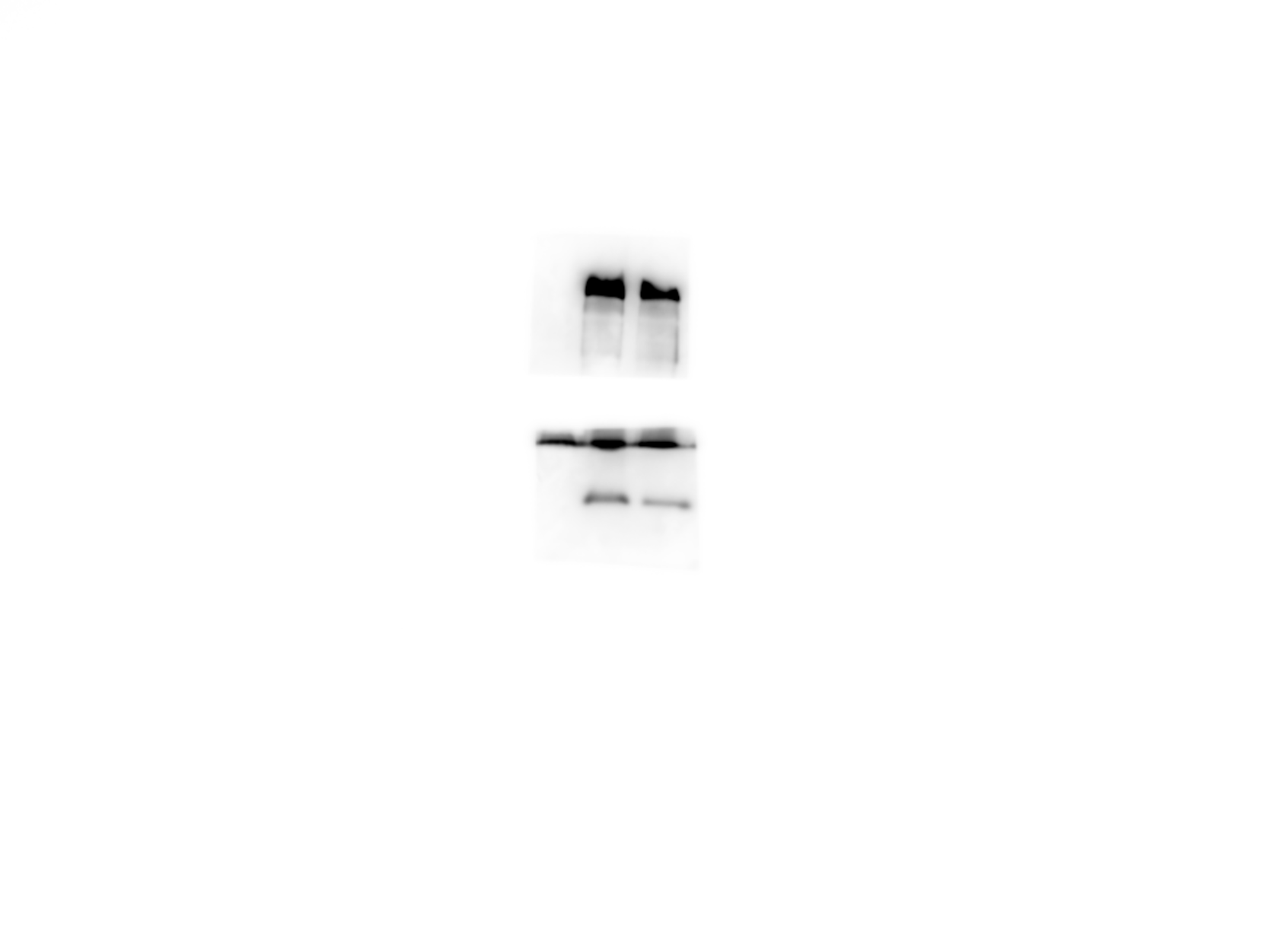

Supplement: Source data 2. [file elife-69269-data2.zip › tiff blots Fig1-7/Fig6B source data 2.tif]

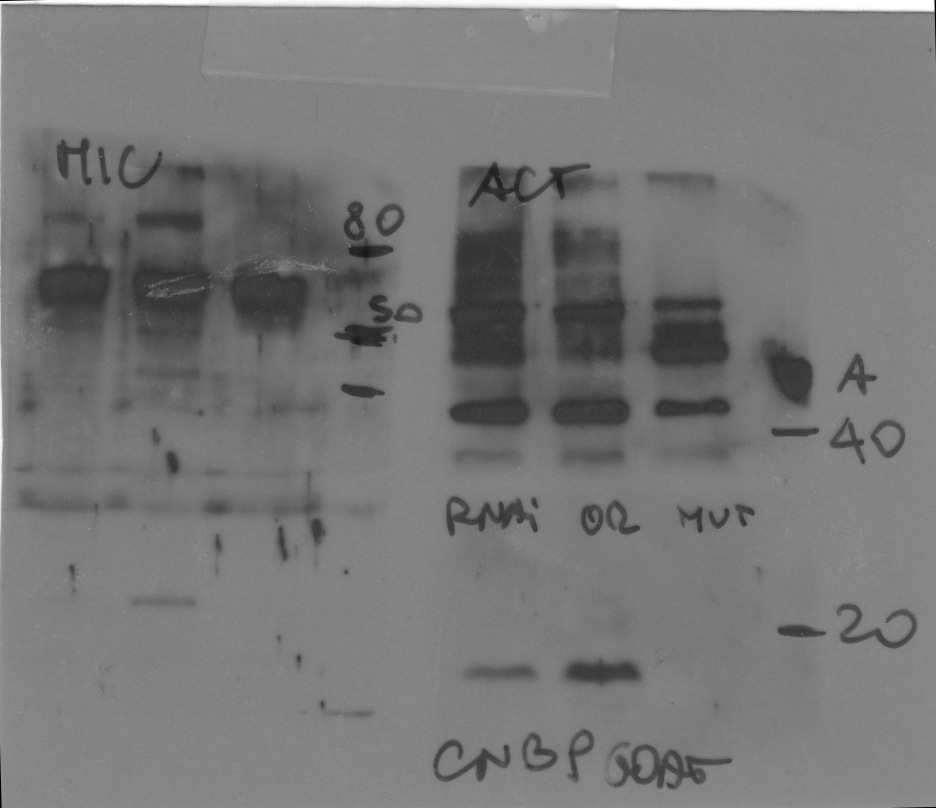

Supplement: Source data 2. [file elife-69269-data2.zip › tiff blots Fig1-7/Fig1E source data 2.jpeg]

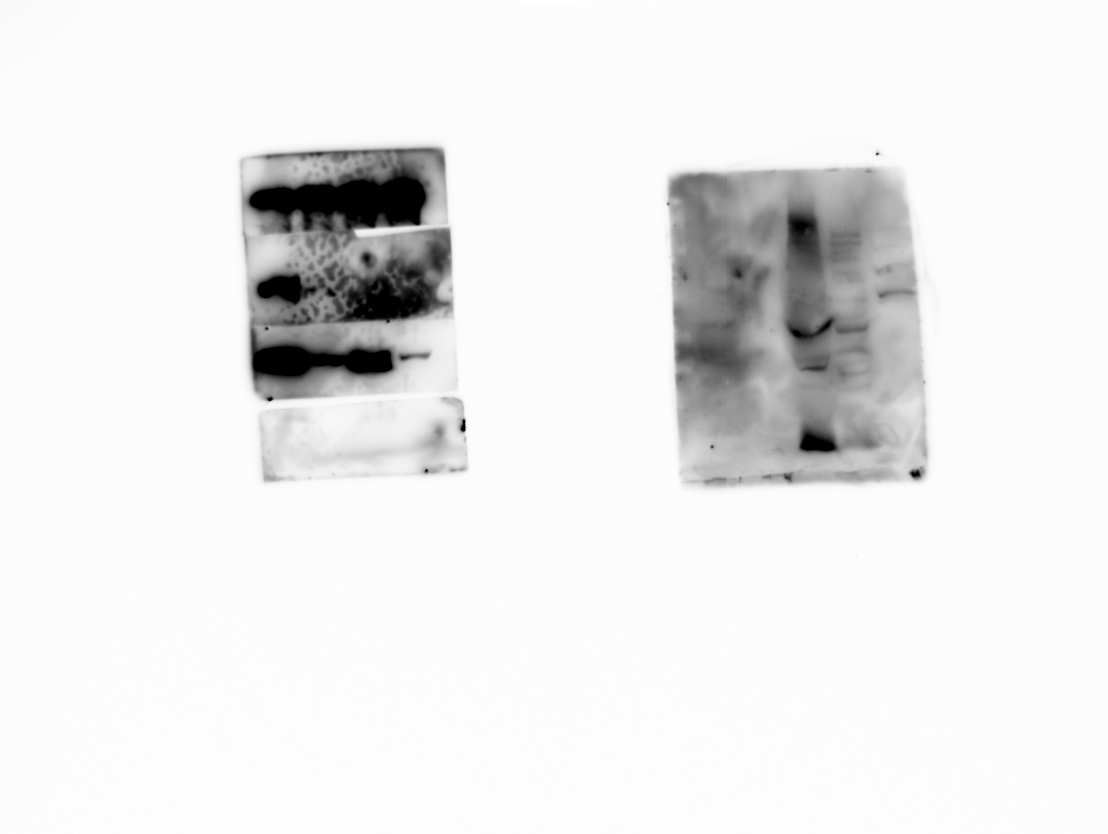

Supplement: Source data 2. [file elife-69269-data2.zip › tiff blots Fig1-7/Fig3-S1A source data 2.tif]

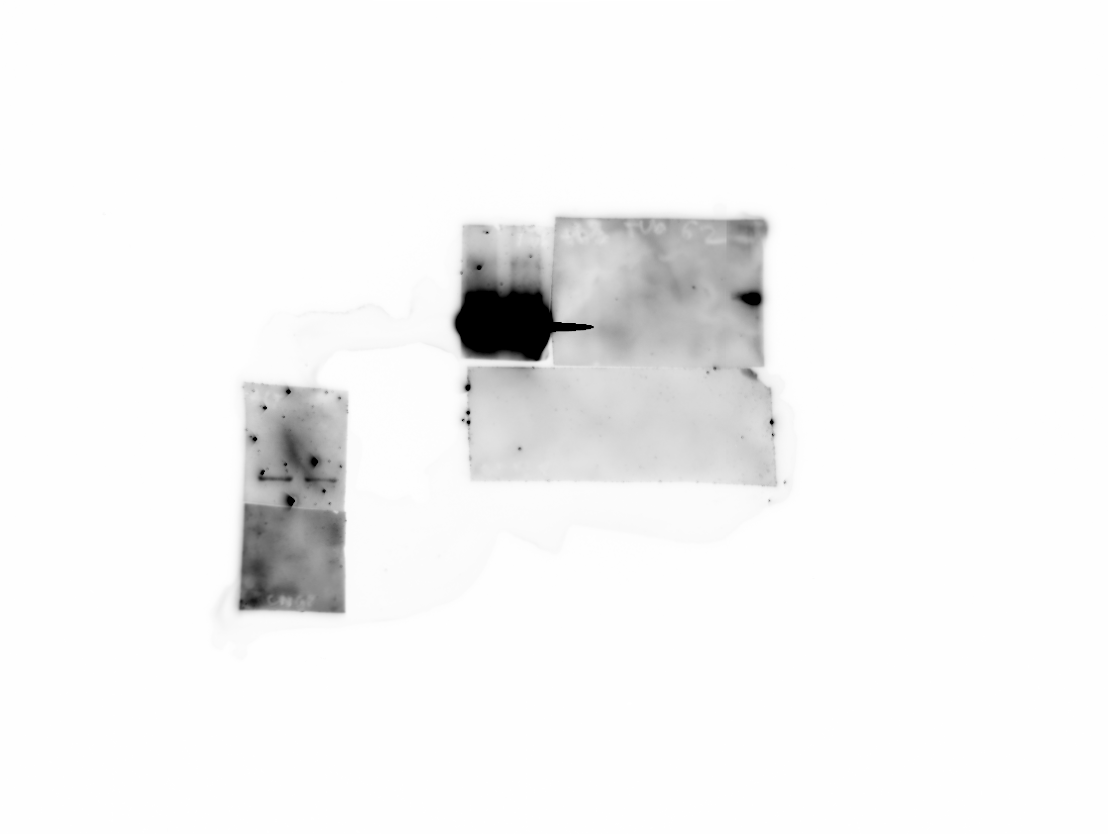

Supplement: Source data 2. [file elife-69269-data2.zip › tiff blots Fig1-7/Fig6-S4A source data 2c.tif]

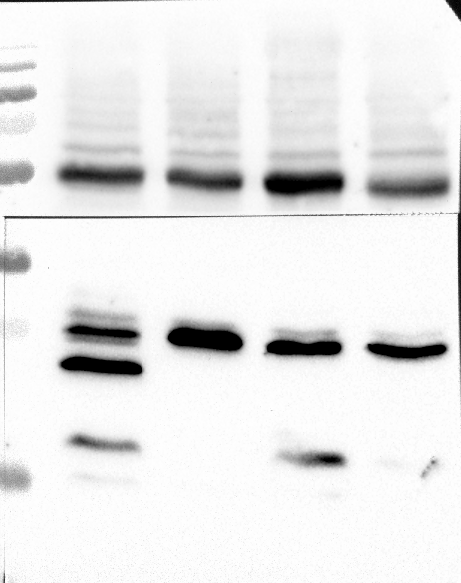

Supplement: Source data 2. [file elife-69269-data2.zip › tiff blots Fig1-7/Fig7-S2 source data.tif]

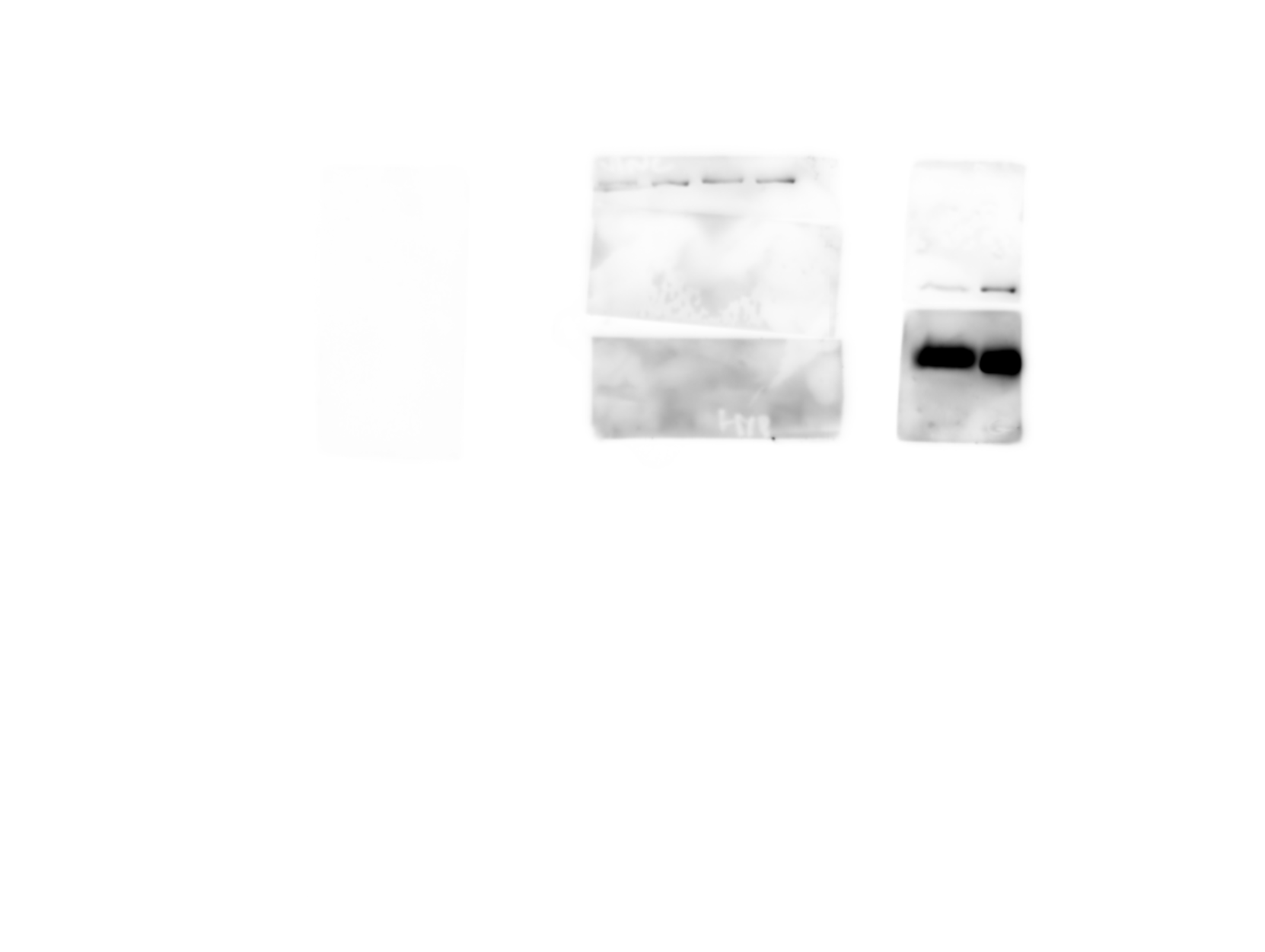

Supplement: Source data 2. [file elife-69269-data2.zip › tiff blots Fig1-7/Fig6-S4A source data 2b.tif]

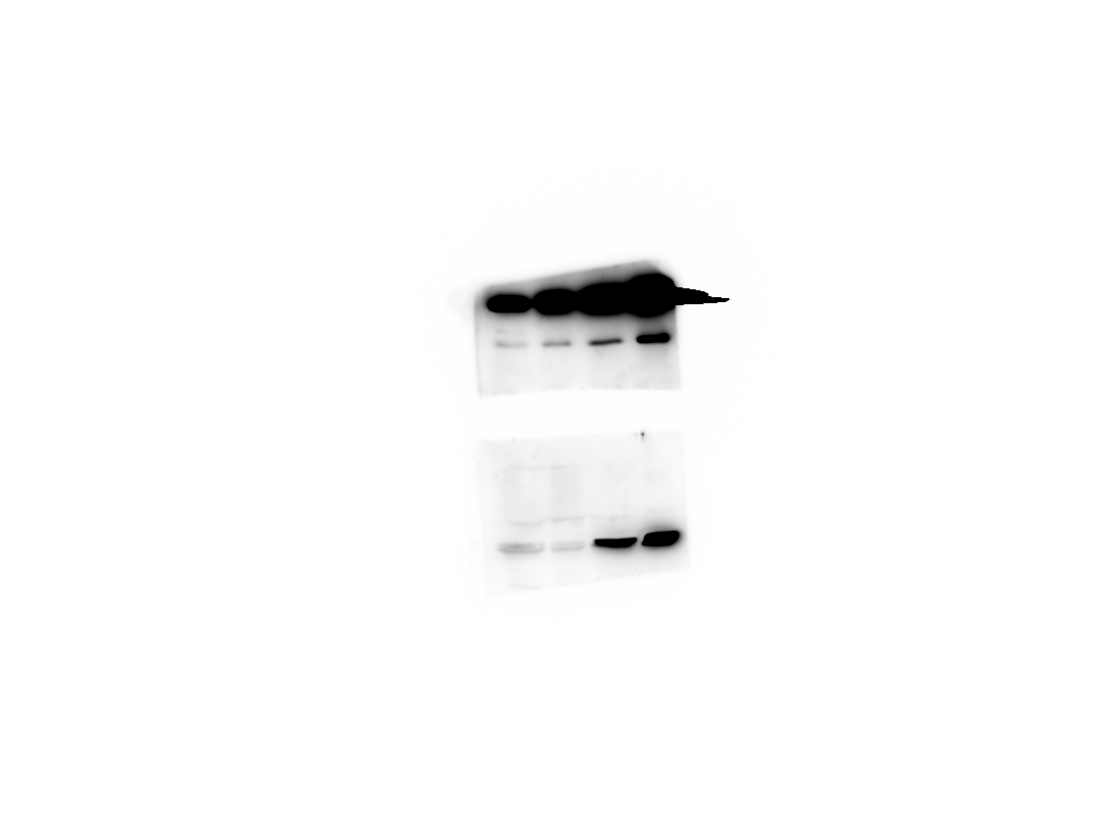

Supplement: Source data 2. [file elife-69269-data2.zip › tiff blots Fig1-7/Fig6-S4A source data 2a.tif]

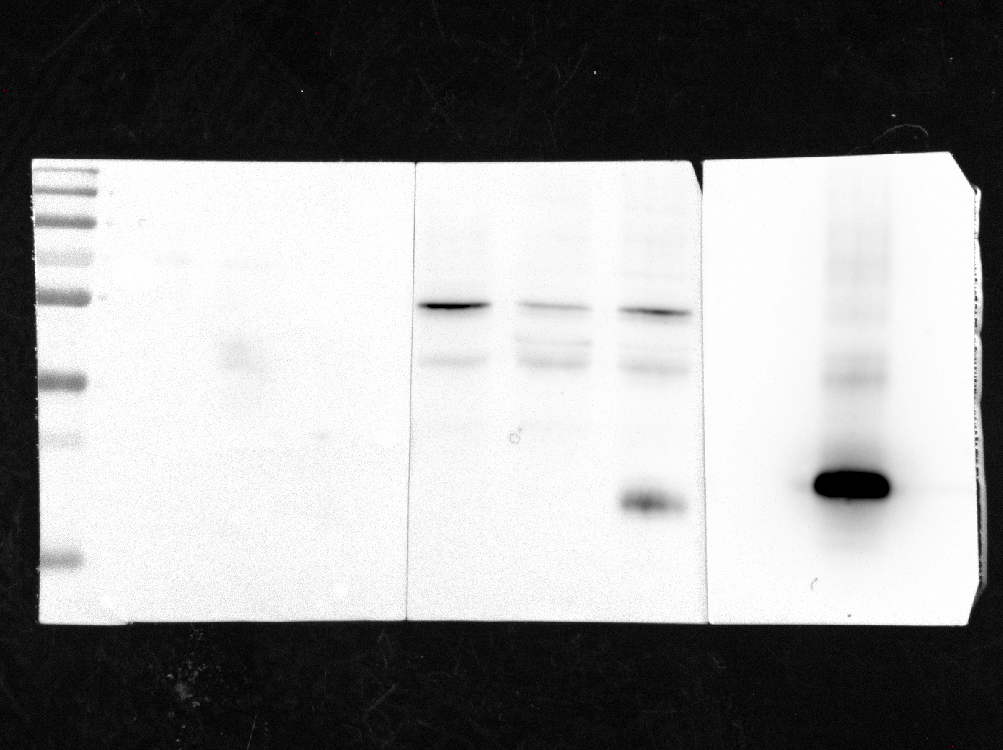

Supplement: Source data 2. [file elife-69269-data2.zip › tiff blots Fig1-7/Fig2B source data 2.tif]

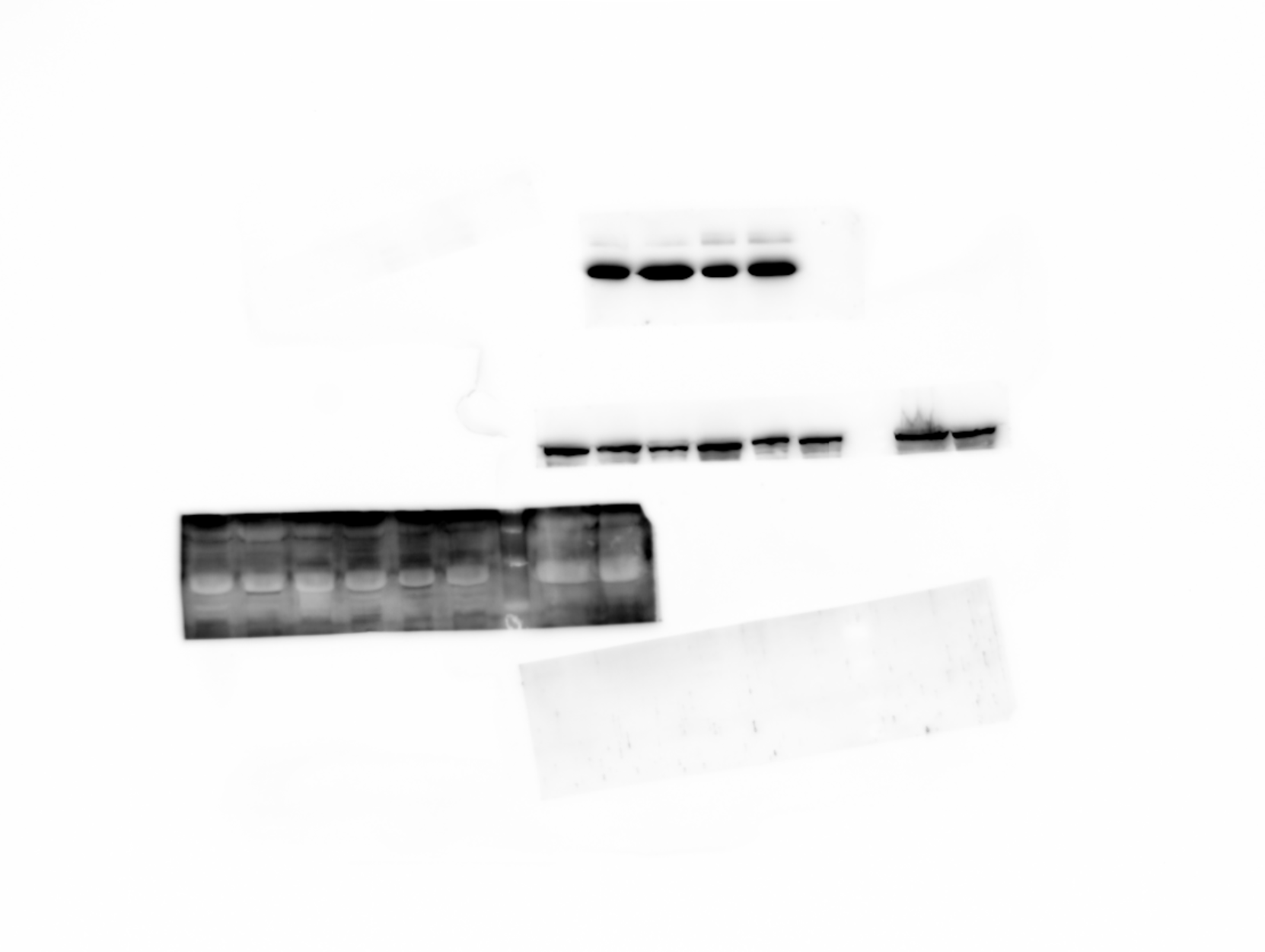

Supplement: Source data 2. [file elife-69269-data2.zip › tiff blots Fig1-7/Fig5A source data 2b.tif]

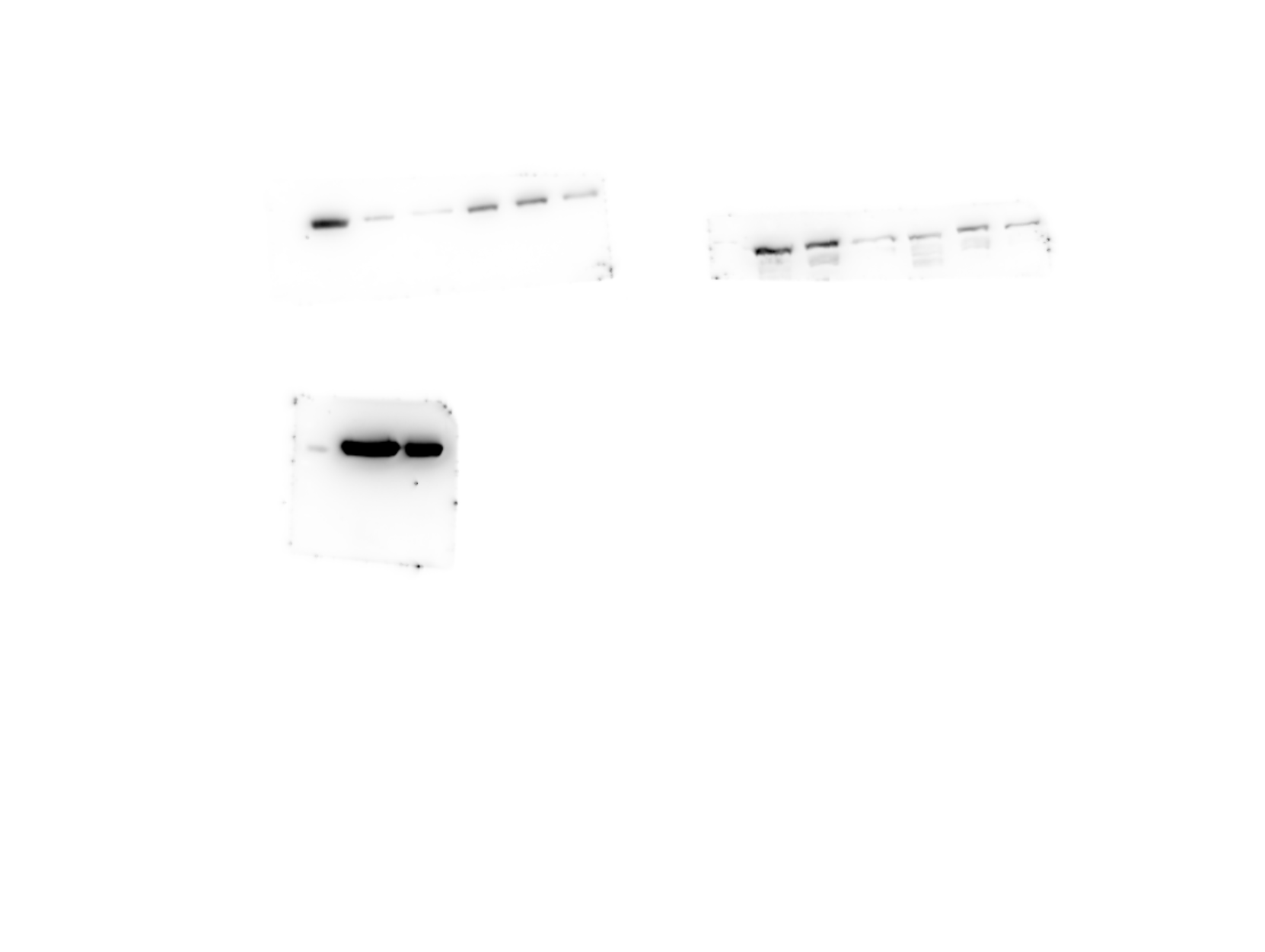

Supplement: Source data 2. [file elife-69269-data2.zip › tiff blots Fig1-7/Fig5A source data 2c.tif]

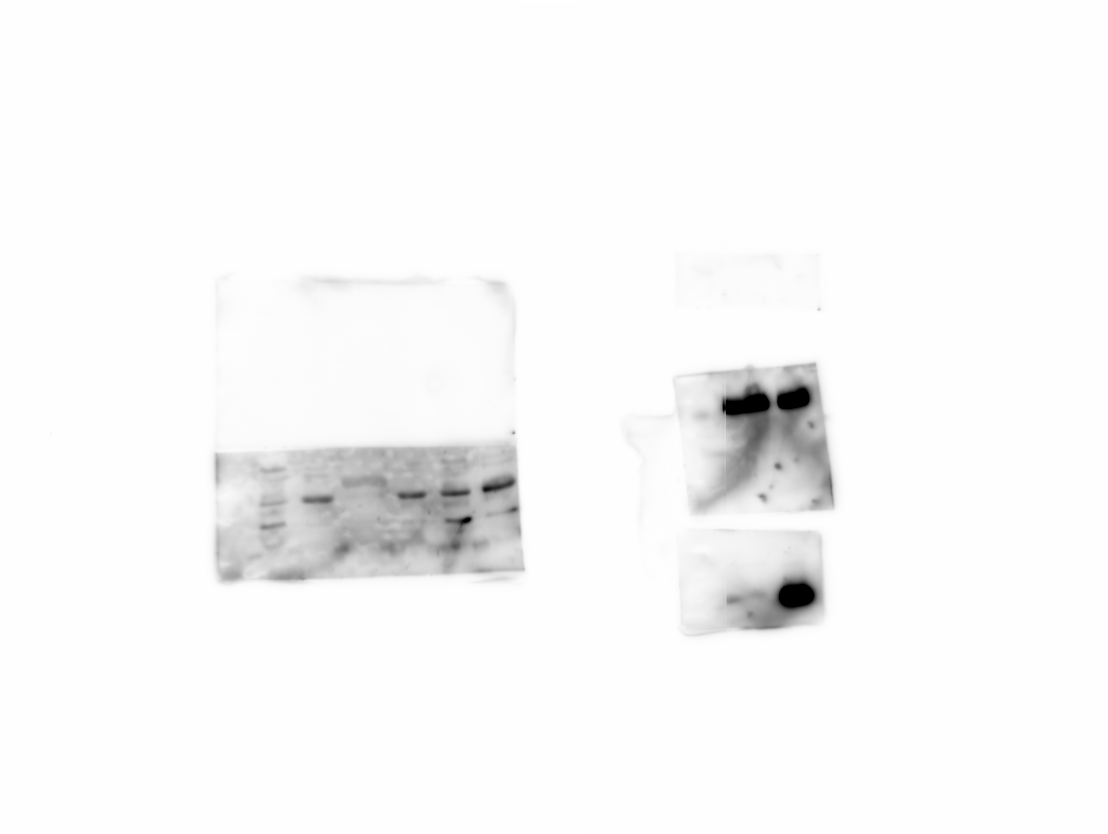

Supplement: Source data 2. [file elife-69269-data2.zip › tiff blots Fig1-7/Fig5A source data 2a.tif]

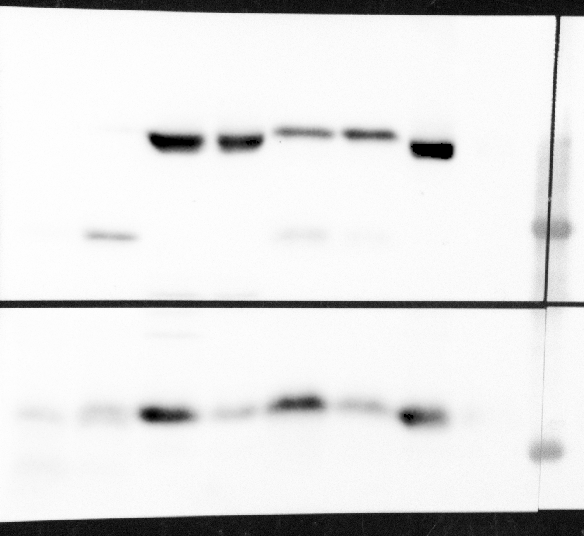

Supplement: Source data 2. [file elife-69269-data2.zip › tiff blots Fig1-7/Fig3A source data 2a.tif]

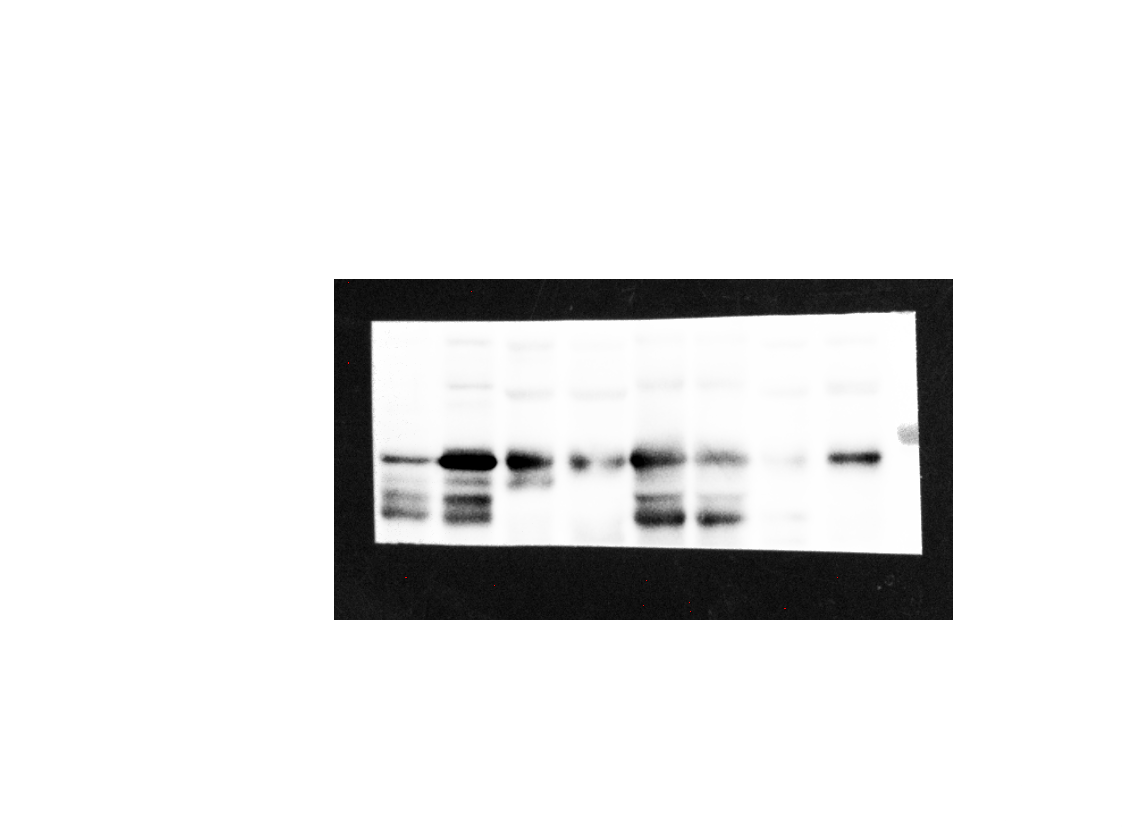

Supplement: Source data 2. [file elife-69269-data2.zip › tiff blots Fig1-7/Fig3A source data 2b.tif]
